# Validation of Recombinant Chicken Liver Bile Acid Binding Protein as a Tool for Cholic Acid Hosting

**DOI:** 10.3390/biom11050645

**Published:** 2021-04-27

**Authors:** Giusy Tassone, Maurizio Orlandini, Massimo Olivucci, Cecilia Pozzi

**Affiliations:** 1 Department of Biotechnology, Chemistry and Pharmacy, University of Siena, Via Aldo Moro 2, 53100 Siena, Italy; giusy.tassone@unisi.it (G.T.); maurizio.orlandini@unisi.it (M.O.); massimo.olivucci@unisi.it (M.O.); 2 Department of Chemistry, Bowling Green State University, Bowling Green, OH 43403, USA

**Keywords:** fatty acid-binding protein, chicken liver bile acid-binding protein, bile acids, cholic acid, X-ray crystallography, high-resolution crystal structure

## Abstract

Bile acids (BAs) are hydroxylated steroids derived from cholesterol that act at the intestinal level to facilitate the absorption of several nutrients and also play a role as signaling molecules. In the liver of various vertebrates, the trafficking of BAs is mediated by bile acid-binding proteins (L-BABPs). The ability to host hydrophobic or amphipathic molecules makes BABPs suitable for the distribution of a variety of physiological and exogenous substances. Thus, BABPs have been proposed as drug carriers, and more recently, they have also been employed to develop innovative nanotechnology and biotechnology systems. Here, we report an efficient protocol for the production, purification, and crystallization of chicken liver BABP (cL-BABP). By means of target expression as His^6^-tag cL-BABP, we obtained a large amount of pure and homogeneous proteins through a simple purification procedure relying on affinity chromatography. The recombinant cL-BABP showed a raised propensity to crystallize, allowing us to obtain its structure at high resolution and, in turn, assess the structural conservation of the recombinant cL-BABP with respect to the liver-extracted protein. The results support the use of recombinant cL-BABP for the development of drug carriers, nanotechnologies, and innovative synthetic photoswitch systems.

## 1. Introduction

**Figure 1:**
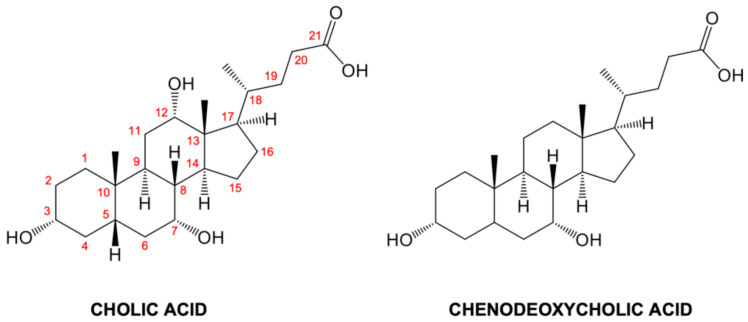
Chemical structures of cholic acid (CA) and chenodeoxycholic acid (CDCA). The CA atom numbering is indicated.

Bile acids (BAs) are hydroxylated steroids synthesized by liver cells via a complex multistep process relying on the cytochrome P450-mediated oxidation of cholesterol [1,2]. BAs are involved in important physiological functions as they facilitate the absorption of various intestinal nutrients (e.g., lipids and vitamins) and promote the biliary secretion of lipids, toxic metabolites, and xenobiotics [1,2]. In humans, the main primary BAs produced by liver cells are cholic acid (CA) and chenodeoxycholic acid (CDCA) (Figure 1) [1,2]. To promote nutrient digestion and absorption in the digestive tract, CA and CDCA are conjugated with either taurine or glycine, generating bile salts that form mixed micelles with phospholipids and cholesterol [1,2,3]. BAs also act as signaling molecules and metabolic regulators through the activation of nuclear receptors, such as farnesoid-X-receptors (FXR), liver-X-receptor (LXR), and G protein-coupled receptors (GPCR), controlling enterohepatic circulation and maintaining the metabolic homeostasis [4]. 

The trafficking of BAs is mediated by intracellular lipid-binding proteins (iLBPs), soluble carriers characterized by an internal cavity hosting hydrophobic or amphipathic molecules [5,6]. The iLBP family is composed of various phylogenetically related, low molecular weight proteins including cellular retinol-binding proteins (CRBPs), retinoic acid-binding proteins (CRABPs), and fatty acid-binding proteins (FABPs) [7,8,9,10,11,12,13,14]. These proteins share a common structural architecture consisting of a ten-stranded antiparallel β-barrel and two α-helices enclosing the internal cavity where hydrophobic ligands bind [15,16,17,18,19,20,21]. Among iLBPs, FABPs play a key role as carriers of fatty acids and other lipophilic ligands, being involved in their solubilization, storage, and intra- and transcellular trafficking. Although the physiological functions involving them are known, the processes of loading and releasing of guest molecules are not yet fully understood [19]. A wide variety of FABPs, named according to their source tissue, have been characterized in vertebrate organisms [17,18,20,22,23,24]. In the liver, two paralogous groups of FABPs have been found among different organisms, the liver FABPs (L-FABPs), peculiar of mammals, and the liver bile acid binding proteins (L-BABPs), typical of various vertebrates but absent in mammals [25,26]. Among FABPs, a protein that has been extensively investigated as a potential carrier of both physiological and exogenous substances and for nanotechnological applications is the BABP isolated from chicken liver (cL-BABP) [18,19,27].

Inherited and acquired defects of BA transporters are related to the pathogenesis of various hepatobiliary, intestinal and metabolic disorders [28]. Thus, understanding the mechanisms of BA loading and release could lead to the identification of novel strategies to treat these metabolic diseases. Furthermore, the peculiar ability of FABPs to carry small hydrophobic or amphipathic molecules makes them suitable delivery tools for physiological and exogenous molecules [29]. Indeed, cL-BABP was exploited to generate a novel contrast agent for hepato-specific magnetic resonance imaging (MRI). In this study, cL-BABP was loaded with a gadolinium(III) chelate BA derivative, generating a stable, soluble carrier that can be applied as a contrast agent [30]. In a following work, the loading of this protein with xanthene dyes to obtain innovative optoelectronic devices was reported [31]. Recently, cL-BABP has been employed as a protein scaffold aiming at the development of a photo-inducible rhodopsin mimic system [32]. In this study, a synthetic chromophore mimicking the rhodopsin retinal has been conjugated to cL-BABP, obtaining an innovative photoswitch system [32]. The potential employment of these proteins as host for various substances make them attractive tools for the development of effective and versatile drug carriers, nanotechnologies, and innovative synthetic photoswitch-systems [19,29,30,31,32]. To support the development of such applications, the structural characterization of the designed systems could allow a faster improvement of these technologies. 

For this purpose, we focused on the production of the recombinant cL-BABP and its characterization through X-ray crystallography. Unlike previous reports in the literature [18,33], the protocol developed here is based on the expression of cL-BABP in *E. coli* cells as His^6^-tag protein. By the introduction of this affinity tag, we were able to raise the production yield and simplify the purification procedure. The recombinant cL-BABP showed an increased propensity to crystallize, allowing us to obtain its structure at high resolution. To validate the use of recombinant cL-BABP for structural investigations aimed at the development of innovative systems, our structures of cL-BABP and its complex with CA were compared with those formerly reported on the liver-extracted protein [18]. This comparison highlighted the structural matching among the models, thus supporting the application of the recombinant protein for the development of drug carriers, nanotechnologies, and innovative synthetic photoswitch-systems [19,29,30,31,32].

## 2. Results and Discussion

### 2.1. An Efficient Protocol for Expression and Purification of Recombinant cL-BABP in E. coli

**Figure 2:**
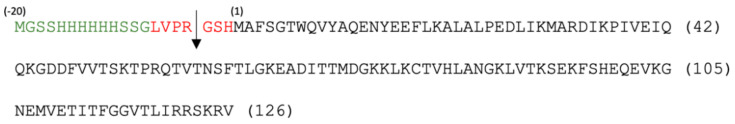
The sequence of His^6^-tag cL-BABP. Amino acids belonging to the His^6^-tag and to the thrombin recognition site are colored green and red, respectively, whereas the sequence of cL-BABP is indicated using black characters. The thrombin cleavage site is indicated by a black arrow. The sequence numbering refers to cL-BABP; thus, the translation start methionine is indicated by 1; negative numbers are used for the amino acids belonging to the removable His^6^-tag.

Different protocols have been previously described to obtain purified cL-BABP both from its natural source, the chicken liver, and from *E. coli* recombinant protein expression [18,27,33]. According to the first reported protocol, cL-BABP was separated from the liver soluble proteins by performing two stages of gel filtration (on Sephadex G-100 and G-50 columns) followed by weak anion exchange chromatography (on DEAE-cellulose column). The yield of this purification procedure was not specified, but in a former study, reporting the extraction of cL-BABP with analogous methods, a final amount of 1.3 g of protein was obtained from about 3 kg of chicken liver [27]. This protein sample was highly homogenous and thus suitable for structural studies by means of X-ray crystallography. More recently, the structural investigation of cL-BABP by NMR spectroscopy was reported using a recombinant protein expressed in *E. coli* [33]. The production protocol relied on protein overexpression induced with 0.7 mM IPTG and by incubating cells overnight at 20 °C. The purification of cL-BABP was then achieved by combining anion exchange (DEAE-cellulose column) and gel filtration chromatography (Sephacryl S-100 HR column). The protein yield reported through this purification procedure was about 90 mg L^−1^ bacterial culture. In order to simplify the purification protocol, minimizing the loss of protein due to multiple purification steps, we cloned the cL-BABP cDNA in an expression vector (pET15b) containing a single His^6^-tag at the protein N-terminus removable by means of thrombin cleavage (Figure 2). To optimize recombinant protein expression, *E. coli* BL21-Gold bacterial cells were chosen, and expression trials were performed by screening two different IPTG concentrations (0.5 and 1 mM), temperatures (25 °C and 32 °C), and incubation intervals (4 and 24 h). The best condition turned out to be 32 °C, 0.5 mM IPTG, and 4-h incubation, which produced the highest amount of protein in the soluble cellular fraction. The isolation of the target was then achieved through a purification procedure relying on two stages of nickel-affinity chromatography by means of a step-gradient elution protocol. After the first stage, a highly pure sample of His^6^-tag-cL-BABP was obtained. The second chromatographic step, performed after tag cleavage by thrombin protease, was done to obtain the pure mature cL-BABP. The final yield achieved through these simple procedures was improved to about 150 mg L^−1^ bacterial culture, allowing us to obtain a large amount of pure and homogeneous protein to perform X-ray crystallographic studies.

### 2.2. Crystallization of cL-BABP and Its Complex with CA

**Figure 3:**
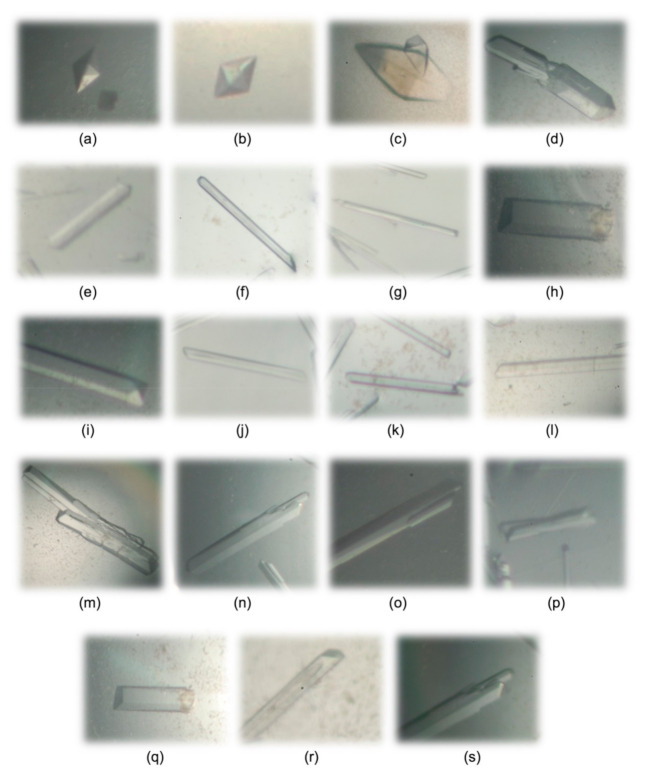
Crystals of cL-BABP (**a**–**r**) and of its CA-complex (**s**) grew using different crystallization conditions (Table 1). These crystals were selected to perform the X-ray crystallographic screening.

The crystallization of cL-BABP was formerly reported using the protein sample extracted from chicken liver cells [18]. According to the results described by the research group of Prof Monaco, attempts to obtain strong diffracting crystals suitable for X-ray analysis under standard gravity conditions were unsuccessful. Thus, they reported the growth of liver-extracted cL-BABP crystals under microgravity (on the International Space Station during the STS-100/ISS 6A mission). To perform this experiment, droplets of 40 microliters were prepared by mixing equal volumes of protein and precipitant (20% PEG6000 and 0.1 M imidazole, pH 7.5) solutions in the High-Density Protein Crystal Growth System. A maximal resolution of 2.0 Å was reported for liver-extracted cL-BABP crystals grown under these microgravity conditions [18]. On the other hand, the crystallization campaign performed by us using the recombinant protein produced in *E. coli* cells resulted in crystal growth using more than 30 different precipitant solutions. At variance with the liver-extracted protein, our recombinant cL-BABP displayed a higher propensity to crystallize. Indeed, crystal growth was observed both at room temperature and at 8 °C in a wide range of pH (spanning from 4.6 to 8.5) and with various combination of salts, organic acids, alcohols, and PEGs (with molecular weights in the range 2000–10,000), as precipitant agents (Figure 3 and Table 1). cL-BABP crystals, grown under these different conditions, displayed various shapes (octahedral, rhombohedral, parallelepipedal, and needle-shaped) and sizes (spanning from microcrystals to rhombohedral crystals having dimensions of about 600 × 600 × 200 µm) (Figure 3 and Table 1). Prior to the X-ray crystallographic screening, eighteen crystallization conditions were selected among all those identified, considering precipitant composition and crystal habitus and dimensions as selection criteria (Table 1). Although few of them showed a low diffraction power, eight cL-BABP crystals, obtained under different conditions, diffracted to resolutions ≤2 Å. The strongest diffraction pattern, having a maximal resolution of 1.4 Å, was observed from parallelepipedal crystals grown at 8 °C using a precipitant solution composed of 20% PEG4000, 20% 2-propanol, and 0.1 M sodium citrate, pH 5.6 (Figure 3o and Table 1).

The same crystallization condition was also applied to obtain crystals of cL-BABP in complex with CA. As for the apo-protein, a simplification of the crystallization procedure was achieved by using the recombinant protein with respect to liver-extracted cL-BABP [18]. The complex with the latter protein was obtained by combining the liver-extracted cL-BABP with a ten-times molar excess of CA and stirring the resulting solution overnight at 20 °C before concentration under nitrogen pressure. The solution of the complex was crystallized under microgravity conditions, as the aforementioned apo-protein. At variance with this procedure, we obtained the formation of the complex just by combining the recombinant cL-BABP with a three-times molar excess of CA and incubating the resulting solution for 1 h at 4 °C prior to the crystallization setup in the laboratory environment. Crystals of the cL-BABP-CA complex showed shapes and sizes comparable to those of the recombinant apo-protein (Figure 3s). 

The high crystallization propensity of recombinant cL-BABP allows obtaining strong diffracting crystals also under standard laboratory conditions, making it a suitable tool for structural and functional studies.

### 2.3. Structural Analysis of the Recombinant cL-BABP

**Figure 4:**
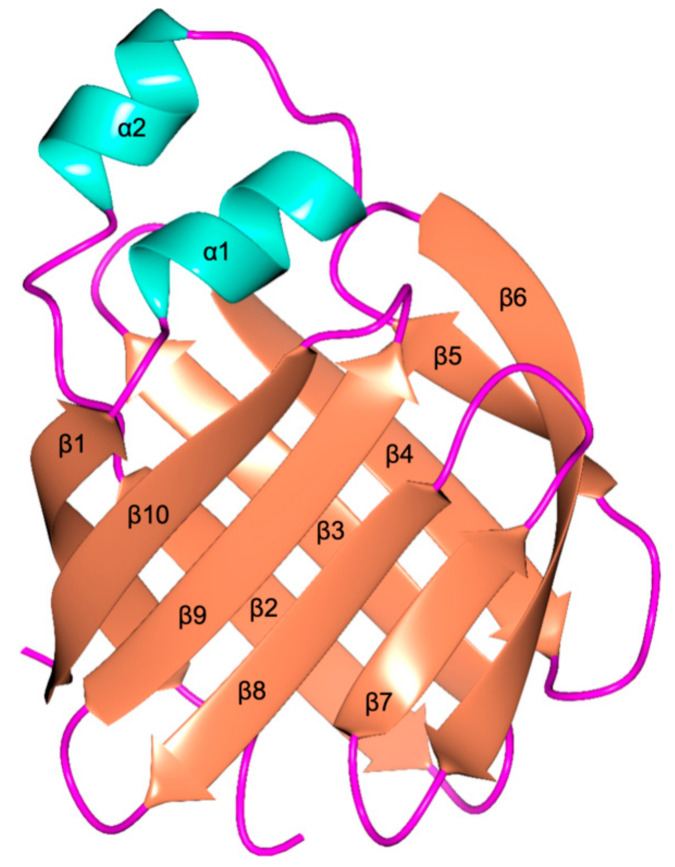
Cartoon representation of the tertiary structure of cL-BABP. α-helices are colored in coral, β-sheets in cyan, and loops in magenta.

The structure of cL-BABP was obtained to 1.40 Å resolution in the orthorhombic space group P2_1_2_1_2_1_. (Table 2). The crystal asymmetric unit is populated by a single protein chain, completely rebuilt in the model. At the N-terminus of the mature cL-BABP, an extension of three amino acids is present due to the His^6^-tag removal by thrombin cleavage (Figure 2). These three residues (numbered from −2 to 0 in Figure 2) are highly flexible, and they were not modeled in the structure. The overall fold of the protein consists of two five-stranded β-sheets (β1–β5 and β6–β10) forming an orthogonal β-barrel (Figure 4). The neighboring strands are interconnected by short reverse turns apart for β1 and β2 that are connected by two α-helices (α1 and α2). The β-barrel encloses the internal ligand-binding cavity, being about 10.0 Å wide and about 13.0 Å in depth. The internal surface of the cavity is mainly hydrophobic, but some hydrophilic residues are placed in an optimal position to interact with the polar functions on the carrier molecules. In the structure of the apo cL-BABP, these residues are involved in a network of hydrogen bonds with ordered water molecules inside the cavity. 

**Figure 5:**
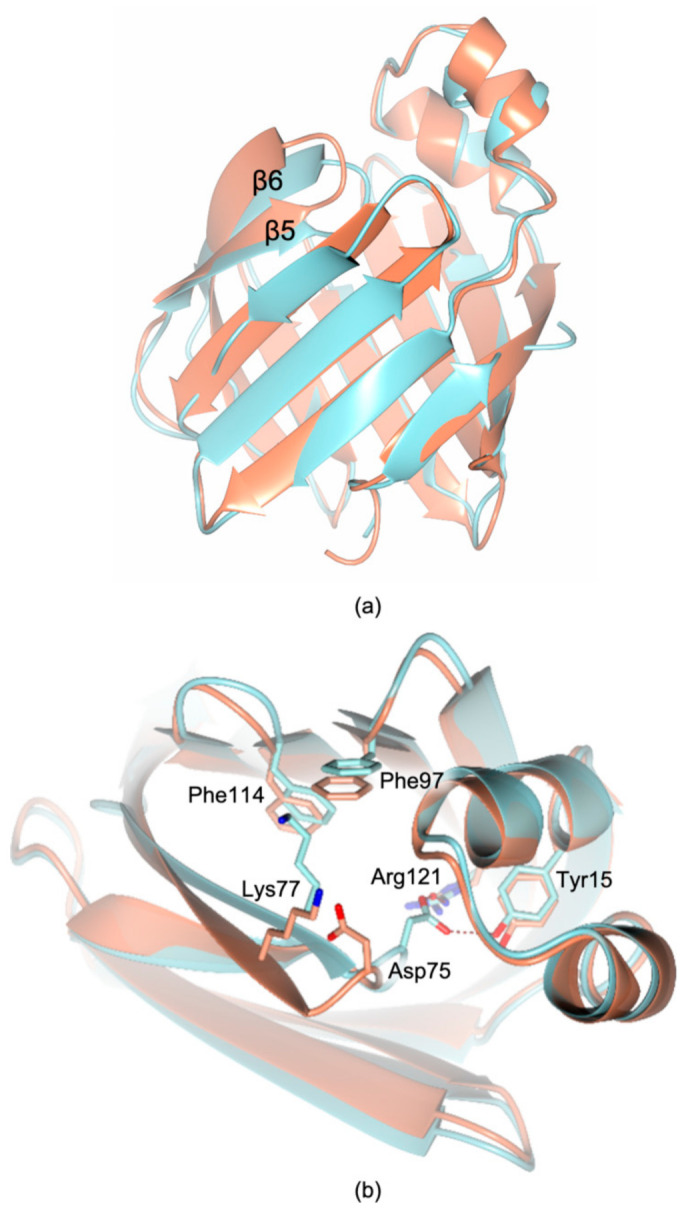
(**a**) Comparison between the structural models of recombinant cL-BABP (coral cartoon) and the chicken liver extracted protein (cyan cartoon; PDB code 1TVQ [18]). Meaningful changes are observed in the loop connecting the strands β5 and β6, where the maximum displacement is reported upon Cα matching. (**b**) View of the ligand cavity in the superimposition between the structural models of recombinant cL-BABP (coral cartoon and carbons) and the chicken liver extracted protein (cyan cartoon and carbons; PDB code 1TVQ [18]). In all figures, hydrogen bonds are displayed as tan dashed lines; oxygen and nitrogen atoms are colored red and blue, respectively.

Former investigations on cL-BABP reported the structural characterization of the protein extracted from chicken liver cells to 2.00 Å resolution, obtaining crystals under microgravity conditions (as discussed in Section 2.2) [18]. The comparison between this lower resolution structure and our model shows a quite conserved protein folding, reporting a rmsd upon Cα matching of 0.8 Å (Figure 5a). The maximal displacement, resulting in 4.24 Å, is observed on Asp75 belonging to the β5-β6 loop. In the structure of the recombinant cL-BABP, the region including amino acids 73–79 is shifted far from the inner cavity leading to a more structured β6 strand which extends from residue 76 to 85 (Figure 5b). On the other hand, in the structure of the liver-extracted protein, both Asp75 and Lys77 point towards the internal cavity hindering this area (Figure 5b). Indeed, the side chain of Asp75 is exposed inside the cavity, interacting either directly or through water molecules with the α1 Tyr15 and with the β10 Arg121. The adjacent residue, Lys77, points instead towards the β8 Phe97 and the β9 Phe114, closing this solvent access area to the internal cavity. 

The apo-state of the recombinant cL-BABP was previously investigated by NMR spectroscopy reporting the characterization of its structural model also through this technique [33]. Although the overall fold is similar to that observed in the structure characterized by X-ray crystallography, the superimposition shows meaningful changes in various protein areas. Indeed, the comparison between the NMR and X-ray structural models of recombinant cL-BABP results in a rmsd value upon Cα matching of 2.7 Å.

### 2.4. Structural Characterization of Recombinant cL-BABP in Complex with CA

**Figure 6:**
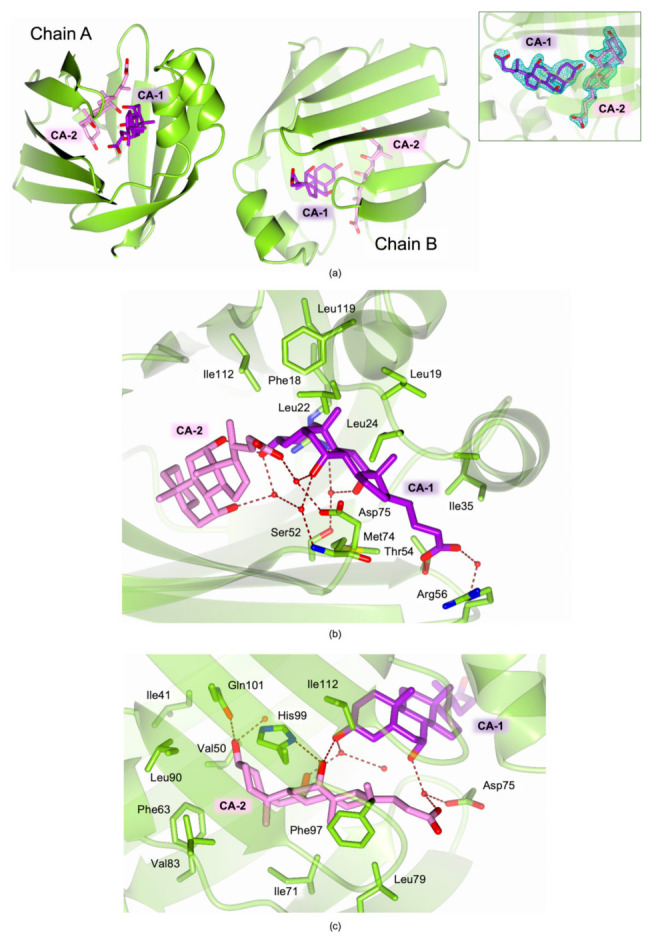
Crystal structure of recombinant cL-BABP (green-yellow cartoon and carbons) in complex with CA. The inner cavity of the protein is populated by two CA molecules, named CA-1 (violet carbons) and CA-2 (pink carbons). (**a**) Cartoon representation of the two cL-BABP chains found in the crystal asymmetric unit. The fitting of the CA molecules in the omit map (teal mash, contoured at the 3.0 σ level) is shown in the inset. (**b**,**c**) Ligand cavity view of (**b**) CA-1 populating the cL-BABP site 1 and (**c**) CA-2 populating the cL-BABP site 2. In all figures, water molecules are displayed as red spheres; sulfur atoms are colored yellow.

The crystal structure of cL-BABP in complex with CA was determined at 1.83 Å resolution, showing high similarity with that formerly reported for the chicken liver-extracted protein in complex with the same molecule (PDB id: 1TW4) [18]. Crystals of the cL-BABP-CA complex belong to the orthorhombic space group P2_1_2_1_2_1,_ and two chains are found in the crystal asymmetric unit (Table 2). The ligand-binding cavity is large enough to accommodate two molecules of CA (named CA-1 and CA-2) in two distinct sites, defined as sites 1 and 2 (Figure 6a). The two CAs are mainly stabilized by van der Waals interactions; nonetheless, their hydroxyl and carboxylate moieties are H-bonded to various inner cavity residues and with each other. CA-1 occupies site 1 lined by residues belonging to the strands β1-β6 and β10, and by the two α-helices (Figure 6b). CA-1 entails extensive van der Waals interactions with Phe18, Leu19, Leu22, Leu24, Ala32, Ile35, Ala69, Ile112, and Leu119. The carboxylate moiety of CA-1 forms a water-mediated interaction with Arg56 and receives H-bonds from Gln57 and Thr54. 

The CA-1 hydroxyls 7 and 12 (atom numbering is displayed in Figure 1) form water-mediated interactions with Asp75 and Met74 and with Arg121 and Ser52, respectively. On the other hand, CA-2 is accommodated in the second site, lined by residues of the strands β2-β9 (Figure 6c). CA-2 is mainly stabilized in this site by van der Waal interactions entailed with Ile41, Val50, Phe63, Ile71, Leu79, Val83, Leu90, Phe97, and Ile112, nonetheless, its hydroxyl moieties 3 and 12 are H-bonded to Gln101 and His99. The latter CA-2 hydroxyl also forms a water-mediated interaction with the CA-1 hydroxyl 3. Furthermore, the carboxylate moiety of CA-2 receives an H-bond from the CA-1 hydroxyl 7 and forms a water-mediated interaction with Asp75. 

**Figure 7:**
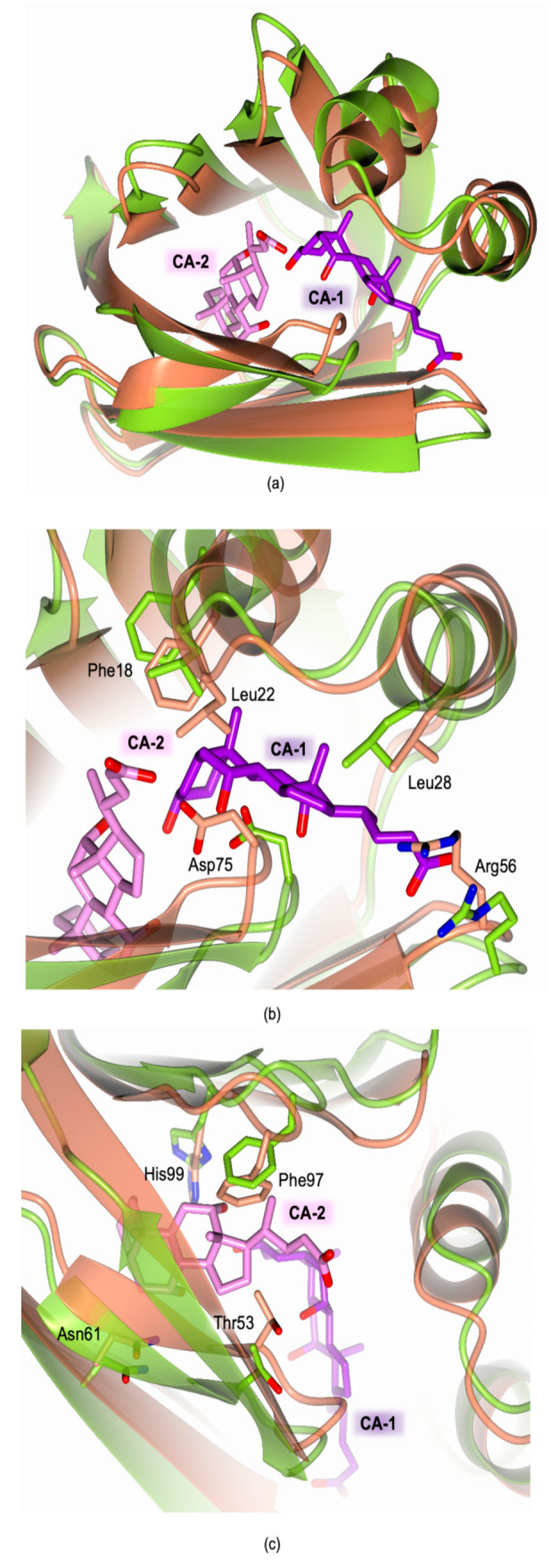
Structural comparison between the recombinant cL-BABP (coral cartoon and carbons) and its complex with CA (green–yellow cartoon and carbons; CA-1 and CA-2 in sticks, violet, and pink carbons, respectively). (**a**) Ligand cavity view showing the aperture occurring upon CA binding, required to accommodate the bile acids molecules. (**b**,**c**) View of the structural rearrangements occurring in the cL-BABP site 1 (**b**) and site 2 (**c**).

The comparison between the structure of the recombinant cL-BABP and its CA-complex evidence that the binding of bile acids inside the cavity does not affect the overall fold of the protein; nonetheless, it occurs by inducing an aperture of the internal cavity due to the slight shifts of various secondary structural elements (Figure 7a). The binding of CA-1 in the first site determines the movement of both helices α1 and α2 and of the loops β5-β6 and β3-β4 (Figure 7b). Indeed, distances of 7.0 Å and 11.1 Å between helix α1 and loop β5-β6 are observed in cL-BABP and in the CA-complex, respectively (distances measured between the Cα atoms of Leu22 and Asp75). This movement allows the shift of Tyr55, Phe18, and Met74, opening the pocket to accommodate CA-1. A further aperture characterizes the external part of the same site showing reciprocal distances of 10.7 Å and 12.4 Å between the helix α2 and the loop β3-β4 in cL-BABP and in the CA-complex, respectively (distances measured between the Cα atoms of Leu28 and Arg56). The movement of the latter loop shifts Arg56 outside the cavity, a rearrangement that is necessary to accommodate the CA-1 carboxylate group. At variance with site 1, the CA-2 binding pocket is more rigid since it is enclosed by the β-barrel (Figure 7c). Even so, the reciprocal distances between β8 and strands β4 and β5 increase to 14.0 Å and 15.3 Å, respectively, in the CA-complex with respect to the apoprotein where distances of 10.2 Å and 12.7 Å are observed (distances measured between the Cα atoms of β8 Phe97, β4 Asn61, and β5 Thr73). This movement determines the shifts of Phe97, His99, Asn61, and Thr73 far from each other, opening the cavity to host CA-2. The structural evidence confirms that CA binding occurs upon the opening of the cL-BABP cavity, as further suggested by molecular dynamics (MD) simulations [18,31,34]. MD data suggested a prominent role of the fluctuation of the loop β5-β6 to accommodate both CA molecules inside the cavity. Indeed, in the structure of the apo-state of cL-BABP extracted from its natural source, the residues belonging to this loop hinder the CA-binding cavity [18]. At variance with this structure, we observe a slight aperture of this loop in the recombinant protein, nonetheless, the cavity is still too tight to accommodate the two CA molecules. The structural comparison between cL-BABP and its CA-complex evidence that various protein areas, further the β5-β6 loop, have to shift to widen the cavity enough for hosting both bile acids. 

## 3. Materials and Methods

### 3.1. Cloning

The cDNA coding for cL-BABP in the pET24d(+) vector was kindly provided by Dr. J. Foote (Fred Hutchinson Cancer Research, Seattle, WA, USA). To generate cL-BABP in frame with the His^6^-tag, the cL-BABP cDNA was PCR-amplified using the following pair of primers: forward A249, 5′-GAGAGAGACA’TATGGCTTTCTCTGGCACC-3′ and reverse A250, 5′-GAGAG’GATCCTCAAACACGTTTAGAACGAC-3′. The PCR product was digested and subcloned into the *NdeI*/*BamHI* sites of the pET15b vector. The construct was checked by sequencing.

### 3.2. cL-BABP Expression and Purification

The expression plasmid containing the cDNA coding for cL-BABP (pET15b-cL-BABP) was introduced by thermal shock in the BL21-Gold *E. coli* strain. Expression trials were performed to optimize the protein production by testing different IPTG concentrations (0.5 and 1 mM), temperatures (20 °C and 32 °C), and incubation intervals (4 and 24 h). The expression levels of the target protein were evaluated by SDS-PAGE analysis and compared to determine the conditions resulting in the maximal protein production in the soluble cellular fraction. In the best condition identified, bacterial cells were cultured in LB medium containing 100 mg L^−1^ ampicillin at 32 °C. cL-BABP over-expression was obtained after 4 h of induction at 32 °C in the presence of 0.5 IPTG when the OD_600nm_ reached values between 0.6–0.8. Cells, harvested by centrifugation, were resuspended in buffer A (100 mM NaCl and 20 mM TRIS, pH 7.5) containing 20 mM imidazole and lysozyme (0.4 mg mL^−1^) and disrupted by sonication after 1 h of incubation on ice. The soluble cellular fraction, separated by centrifugation (13,500× *g*, 1 h, 4 °C), was purified by nickel-affinity chromatography (HisTrap FF 5 mL column, GE Healthcare), using a step-gradient elution protocol relying on imidazole as a competitive agent. His^6^-tag cL-BABP elution was achieved at imidazole concentrations ranging from 75 to 250 mM. Fractions containing the target protein, identified by SDS-PAGE, were pooled and extensively dialyzed in buffer A. The His^6^-tag cleavage (Figure 2) was performed during the dialysis by adding thrombin (1 unit mg^−1^ protein) directly inside the dialysis membrane. After 24 h, the tag cleavage was almost complete (>98%, verified by SDS-PAGE analysis), and the mature protein was subjected to a second stage of nickel affinity chromatography. The effective tag removal was confirmed by Western blotting analysis using a HRP-conjugated His-tag monoclonal antibody (Thermo Fisher Scientific, Waltham, MA, USA). The high purity of the mature cL-BABP (Figure 2) obtained from this procedure was confirmed as >98% by SDS-PAGE and MALDI-TOF mass spectrometry analyses. The final yield was estimated to 150 mg L^−1^ of bacterial culture.

### 3.3. Crystallization

The recombinant mature cL-BABP (Figure 2) was concentrated to 25 mg mL^−1^ in 100 mM NaCl and 20 mM TRIS, pH 7.5, and stored at 8 °C until required. Crystallization trials were performed on this protein sample using the vapor diffusion sitting drop technique either at 8 °C or at room temperature [35]. More than 300 different crystallization solutions from commercially available kits of Hampton Research and Jena Bioscience were screened as precipitant solutions. Drops consisting of 2 μL protein solution and 2 μL precipitant were equilibrated against a 200 μL reservoir. Within 12-h incubation at either 8 °C or room temperature, crystal growth was observed in several conditions, reporting crystalline samples of different habitus and size. Among them, we selected eighteen crystallization conditions to perform an X-ray diffraction screening, allowing us to achieve information on the strongest diffracting crystals. Conditions were chosen considering various selection criteria, including precipitant composition, crystal habitus, and dimensions (Figure 3 and Table 1). Prior to flash freezing in liquid nitrogen, crystals were singularly transferred to the cryoprotectant solutions prepared by adding to each precipitant 20% *v*/*v* of either glycerol or ethylene glycol. Through this screening, we were able to identify the conditions yielding the crystals with the strongest diffraction patterns (*vide infra* and Table 1). The best condition was subsequently used for the crystallization of the recombinant cL-BABP in complex with CA. Crystals of this complex were obtained by co-crystallization using the hanging drop vapor diffusion technique at 8 °C. Protein samples for crystallization experiments were prepared by adding 5 mM CA (dissolved in dimethyl sulfoxide, DMSO) to the aforementioned cL-BABP solution and allowing 1 h incubation at 4 °C. Drops were prepared by mixing equal volumes (2 μL) of complex and precipitant (10% *v*/*v* 2-propanol, 20% PEG4000 and 100 mM Hepes, pH 7.5) solution, and equilibrated over a 500 μL reservoir. Crystals, grown within 1 day, were transferred to the cryoprotectant solution (20% *v*/*v* glycerol and 80% *v*/*v* precipitant) and flash-frozen in liquid nitrogen. The characterization of cL-BABP-CA complex was also attempted through the soaking technique by exposing pre-formed protein crystals to the ligand. 0.25 μL of a 10 mM solution of CA was added directly into the crystallization drop (final ligand concentration of ~1 mM) and crystals were frozen in liquid nitrogen after a 2-h exposure. 

### 3.4. Data Collection, Structure Solution, and Refinement

All crystals of cL-BABP and its complex with CA were screened for diffraction using synchrotron radiation at the Diamond Light Source (DLS, Didcot, UK) beamline I04. Full datasets were collected at 100 K on the Eiger2 XE 16M detector only on the crystal showing the strongest diffraction pattern (Table 1). Reflections were integrated using XDS [36] and scaled with SCALA [37] from the CCP4 suite [37,38]. Molecular replacement was performed using the software MOLREP [39]. The structure of the liver-extracted cL-BABP (PDB code 1TVQ [18], excluding non-protein atoms and water molecules) was used as a research model for the rotation and translation functions. The two structures were refined using REFMAC5 [40] from the CCP4 suite. The molecular graphic software Coot [41] was used for manual rebuilding and modeling of missing atoms in the electron density and to add solvent molecules. In the final refinement cycles of the cL-BABP structure, all atoms were refined anisotropically (except for those with partial occupancies). On the other hand, the structure of cL-BABP–CA complex was refined using the TLS parametrization [42]. The optimal partitioning scheme for the polypeptide chain was calculated through the TLS *Motion Determination* web server [43], resulting in twelve continuous TLS segments. In the structure of the CA complex, the inspection of the Fourier difference map clearly evidenced the presence of two molecules inside the ligand-binding cavity that were modeled accordingly (inset of Figure 6a). The occupancy of the exogenous ligands was adjusted and refined to values resulting in atomic displacement parameters close to those of neighboring protein atoms in fully occupied sites. Despite our attempts to obtain the cL-BABP-CA complex by soaking technique, the structural rearrangements required to accommodate both CA molecules inside the cavity (Section 2.4) reasonably prevented the formation of the complex. Indeed, complex formation was achieved only by the co-crystallization method (Section 3.3), whereas the ligand-free state was consistently observed using the soaking technique. The final models were inspected manually and checked with Coot [41] and PROCHECK [44]. Structural figures were generated using the molecular graphic software CCP4mg [45]. Data collection, processing, and refinement statistics are summarized in Table 2. Final coordinates and structure factors were deposited in the Protein Data Bank (PDB) under the codes 7O0J (recombinant cL-BABP) and 7O0K (recombinant cL-BABP-CA complex).

## 4. Conclusions

In this investigation, we report novel and effective protocols for the expression, purification, and crystallization of recombinant cL-BABP. By means of target expression as His^6^-tag cL-BABP we were able to improve the production yield to about 150 mg L^−1^ obtaining a large amount of pure and homogeneous protein through a simple purification procedure relying only on affinity chromatography. This protocol speeds up and simplifies the procedures for obtaining cL-BABP with respect to the extraction from liver cells and to former reports on recombinant protein expression in *E. coli* bacterial host [18,33]. The recombinant cL-BABP produced with our protocol showed a high propensity to crystallize, allowing us to observe crystal growth in a wide variety of conditions. Furthermore, we were able to improve the resolution of the structural model determined by X-ray crystallography to 1.4 Å. The superimposition between the structures of cL-BABP and its CA-complex highlights the rearrangements required to accommodate the two bile acids in their inner hosting cavity. It is worth noting that the comparison with the former models reported on the liver-extracted protein [18] shows high structural conservation. The validation of the structure allows the reliable design of further application of recombinant cL-BABP for various purposes, such as the development of drug carriers, nanotechnologies, and innovative synthetic photoswitch-systems [19,29,30,31,32].

## Figures and Tables

**Table 1:** Precipitant compositions and crystallization conditions applied to obtain the eighteen cL-BABP crystal samples selected for the X-ray crystallographic screening.

Composition of Precipitant Solutions (Solution Number in Crystallization Kits from Hampton Research and Jena Bioscience) *	Temperature	Crystal Picture	Crystal Habitus	Resolution in X-Ray Diffraction Screening (Å)
2.1 M DL-malic acid pH 7.0 (23 Index–HR)	R.T.	Figure 3a	Octahedral	2.14
2.4 M sodium malonate pH 7.0 (27 Index–HR)	8 °C	Figure 3b	Octahedral	2.32
0.1 M BIS-TRIS pH 5.5, 25% *w*/*v* PEG 3350 (42 Index–HR)	R.T.	Figure 3c	Rhombohedral	2.10
0.1 M Tris pH 8.5, 25% *w*/*v* PEG 3350 (45 Index–HR)	8 °C	Figure 3d	Parallelepipedal	>2.50
0.2 M sodium chloride, 0.1 M HEPES pH 7.5, 25% *w*/*v* PEG 3350 (72 Index–HR)	8 °C	Figure 3e	Parallelepipedal	2.13
0.2 M lithium sulfate, 0.1 M BIS-TRIS pH 6.5, 25% *w*/*v* PEG 3350 (75 Index–HR)	8 °C	Figure 3f	Needle -shaped	1.47
0.2 M ammonium acetate, 0.1 M BIS-TRIS pH 6.5, 25% *w*/*v* PEG 3350 (79 Index–HR)	8 °C	Figure 3g	Needle -shaped	2.07
0.2 M ammonium acetate, 0.1 M HEPES pH 7.5, 25% *w*/*v* PEG 3350 (80 Index–HR)	8 °C	Figure 3h	Parallelepipedal	2.13
0.15 M potassium bromide, 30% *w*/*v* PEGme 2000 (96 Index–HR)	8 °C	Figure 3i	Parallelepipedal	>2.50
0.2 M potassium chloride, 20% *w*/*v* PEG 3350 (8 PEG-Ion–HR)	8 °C	Figure 3j	Needle -shaped	1.88
0.2 M sodium thiocyanate, 20% *w*/*v* PEG 3350 (13 PEG-Ion–HR)	8 °C	Figure 3k	Needle -shaped	1.94
0.2 M ammonium formate, 20% *w*/*v* PEG 3350 (23 PEG-Ion–HR)	8 °C	Figure 3l	Needle -shaped	1.92
0.2 M potassium acetate, 20% *w*/*v* PEG 3350 (29 PEG-Ion–HR)	8 °C	Figure 3m	Parallelepipedal	>2.50
0.1 M HEPES pH 7.5, 1.4 M sodium citrate (38 CS–HR)	8 °C	Figure 3n	Parallelepipedal	2.00
20% *w*/*v* PEG 4000, 20% v/v 2-propanol, 0.1 M tri-sodium citrate pH 5.6 (C2 JBSB-2–JB)	8 °C	Figure 3o	Parallelepipedal	1.40
20% *w*/*v* PEG 4000, 10% v/v 2-propanol 0.1 M HEPES pH 7.5 (C3 JBSB-2–JB)	8 °C	Figure 3p	Parallelepipedal	1.50
30% *w*/*v* PEG 4000, 0.1 M sodium acetate pH 4.6, 0.2 M ammonium acetate (C6 JBSB-2–JB)	8 °C	Figure 3q	Parallelepipedal	1.61
20% *w*/*v* PEG 10,000, 0.1 M HEPES pH 7.5 (E2 JBSB-3–JB)	8 °C	Figure 3r	Parallelepipedal	>2.50

* Index-HR: Index-HR2-144 from Hampton Research; PEG-Ion–HR: PEG/Ion Screen-HR2-126 from Hampton Research; CS-HR: Crystal Screen-HR2-110 from Hampton Research; JBSB-2-JB: JBScreen Basic 2 from Jena Bioscience; JBSB-3-JB: JBScreen Basic 3 from Jena Bioscience.

**Table 2:** Data collection and refinement statistics. Values for the outer shell are given in parentheses.

	cL-BABP	cL-BABP-CA
PDB ID codes	7O0J	7O0K
DATA COLLECTION STATISTICS		
Diffraction source	I04 (DLS)	I04 (DLS)
Wavelength (Å)	0.8920	0.9795
Temperature (K)	100	100
Detector	Eiger2 XE 16M	Eiger2 XE 16M
Crystal-detector distance (mm)	177.2	240.5
Exposure time per image (s)	0.2	0.2
Space group	P2_1_2_1_2_1_	P2_1_2_1_2_1_
No. of subunit in ASU	1	2
*a*, *b*, *c* (Å)	60.68, 62.92, 77.57	39.01, 58.93, 71.29
Resolution range (Å)	71.29–1.40 (1.48–1.40)	62.92–1.83 (1.93–1.83)
Total no. of reflections	306,679 (42,484)	198,330 (28,944)
No. of unique reflections	32,871 (4682)	26,839 (3830)
Completeness (%)	99.3 (98.5)	99.8 (99.6)
Multiplicity	9.3 (9.1)	7.4 (7.6)
(*I*/σ(*I*))	24.3 (3.1)	13.6 (2.4)
*R*_meas_	0.040 (0.688)	0.061 (0.856)
Overall *B* factor from Wilson plot (Å^2^)	18.9	32.2
REFINEMENTS STATISTICS		
Resolution range (Å)	32.55–1.40 (1.44–1.40)	48.87–1.83 (1.88–1.83)
Completeness (%)	99.2 (98.1)	99.7 (99.2)
No. of reflections, working set	31,165 (2238)	25,436 (1834)
No. of reflections, test set	1657 (111)	1326 (86)
Final *R*_cryst_	0.1579 (0.247)	0.2291 (0.337)
Final *R*_free_	0.2065 (0.247)	0.2838 (0.374)
No. of non-H atoms		
Protein	1048	1921
CA	-	116
Water	213	112
Total	1261	2149
R.m.s. deviations Bonds (Å)	0.020	0.012
Angles (°)	2.400	2.140
Average *B* factors (Å^2^)	29.9	40.5
Estimate error on coordinates based on R value (Å)	0.054	0.153
Ramachandran plot		
Most favoured (%)	99.2	98.8
Allowed (%)	0.8	1.2

## Data Availability

Crystal structure final coordinates and structure factors are available in the PDB (www.rcsb.org (accessed on 26 April 2021)) under the codes 7O0J (recombinant cL-BABP) and 7O0K (recombinant cL-BABP-CA complex).
